# Supplementary material for: UPF3A is dispensable for nonsense-mediated mRNA decay in mouse pluripotent and somatic cells
Source: Life Sci Alliance. 2023 Mar 30;6(6):e202201589. doi: 10.26508/lsa.202201589 (PMC10070813; doi:10.26508/lsa.202201589)

Related to Fig 1

Fig 1C

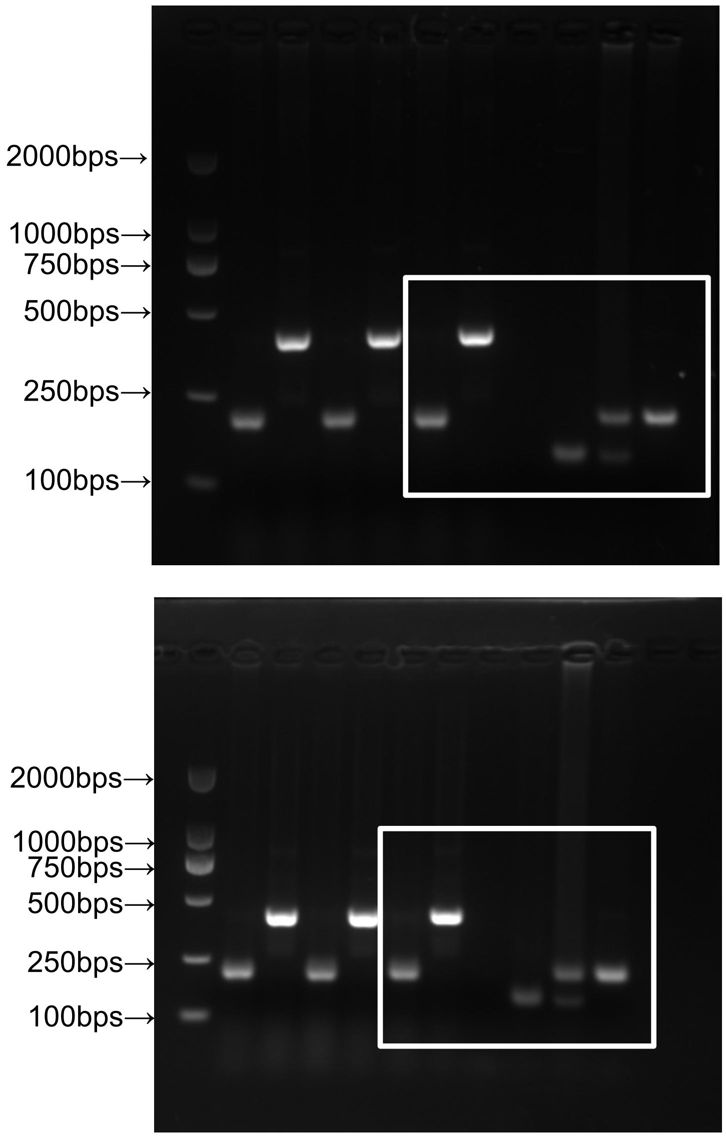

Fig 1D

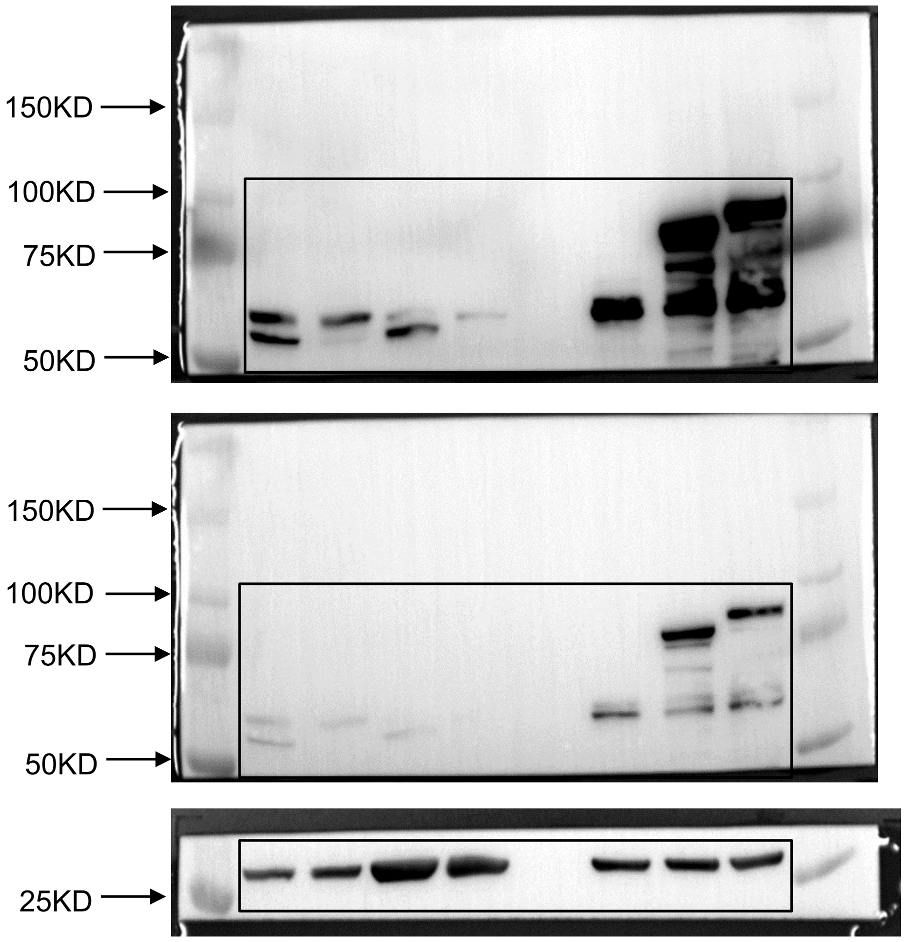

Related to Fig 3

Fig 3A

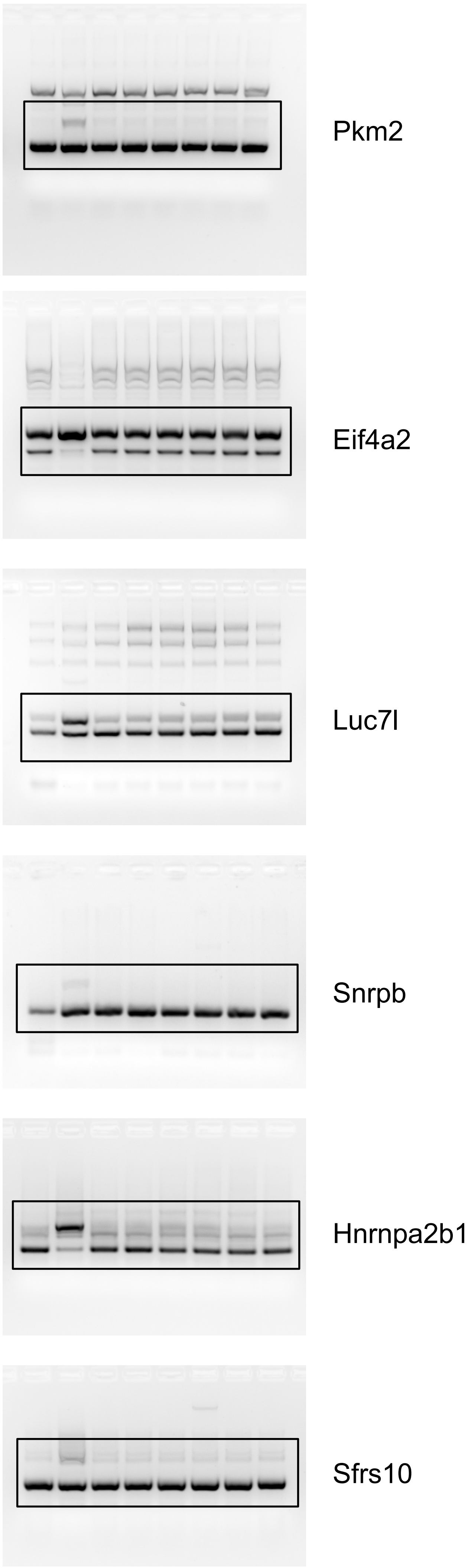

Fig 3B

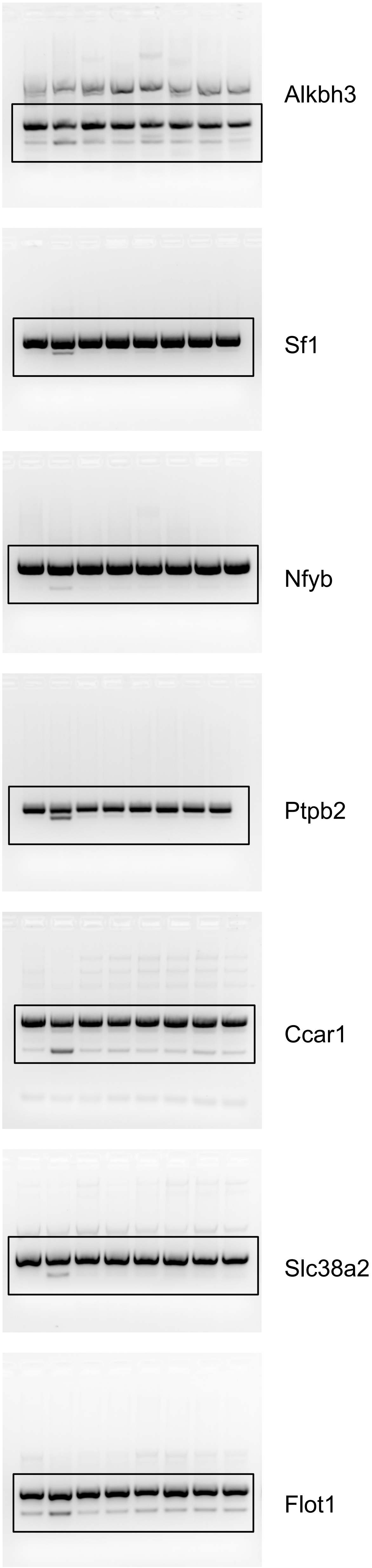

Related to Fig 3

Fig 3C

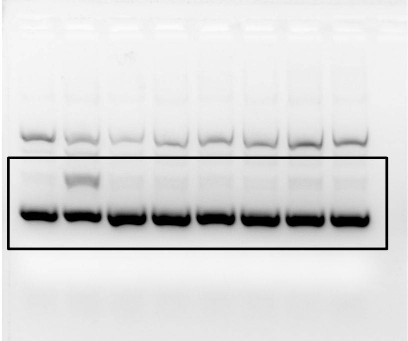

Pkm2

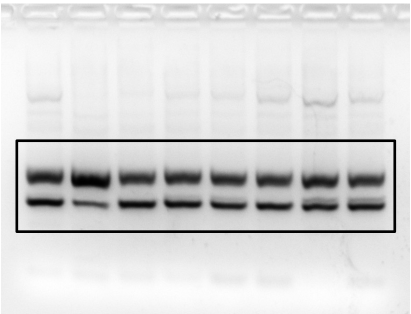

Eif4a2

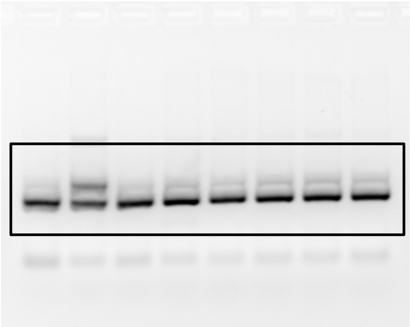

Luc7l

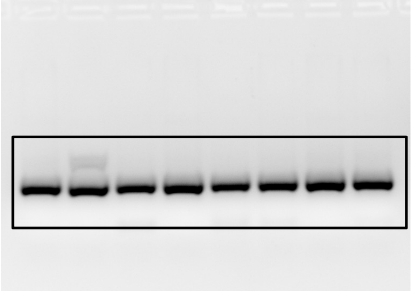

Snrpb

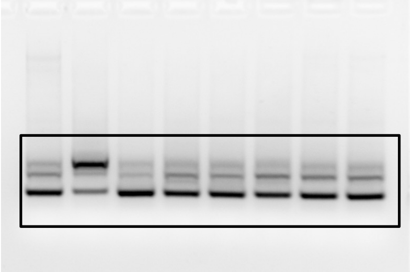

Hnrnpa2b1

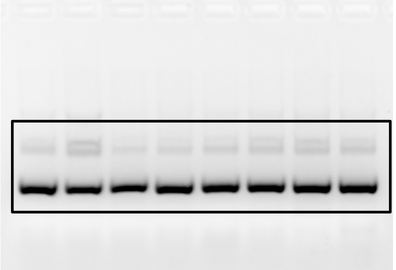

Sfrs10

Fig 3D

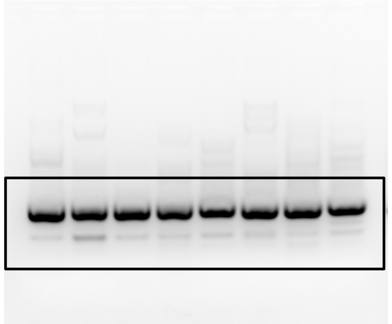

Alkbh3

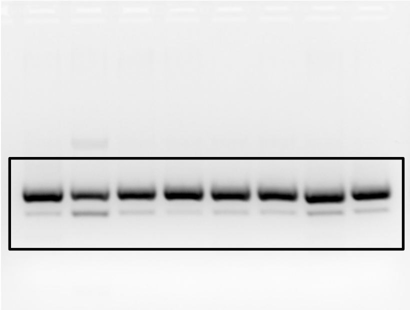

Sf1

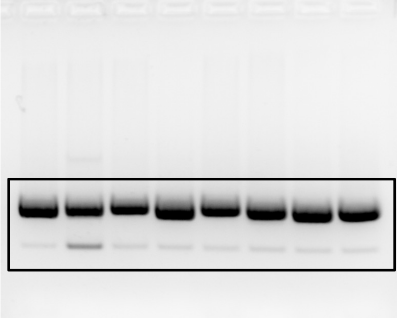

Nfyb

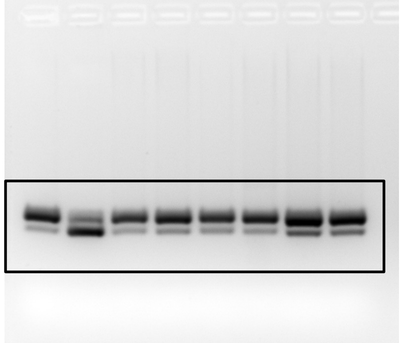

Ptpb2

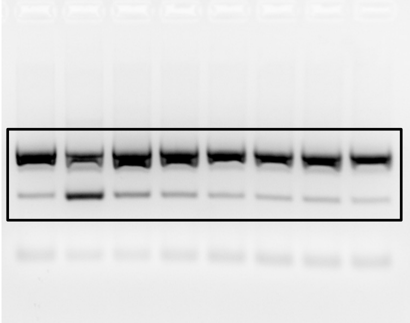

Ccar1

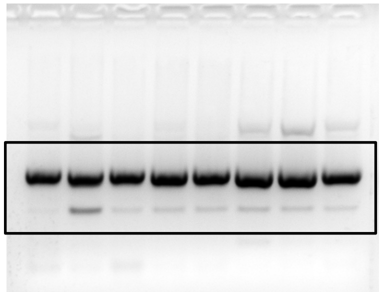

Slc38a2

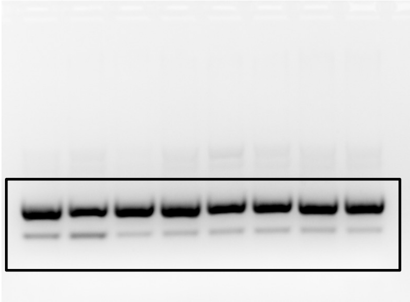

Flot1

Related to Supp Fig 1

Supp Fig 1B

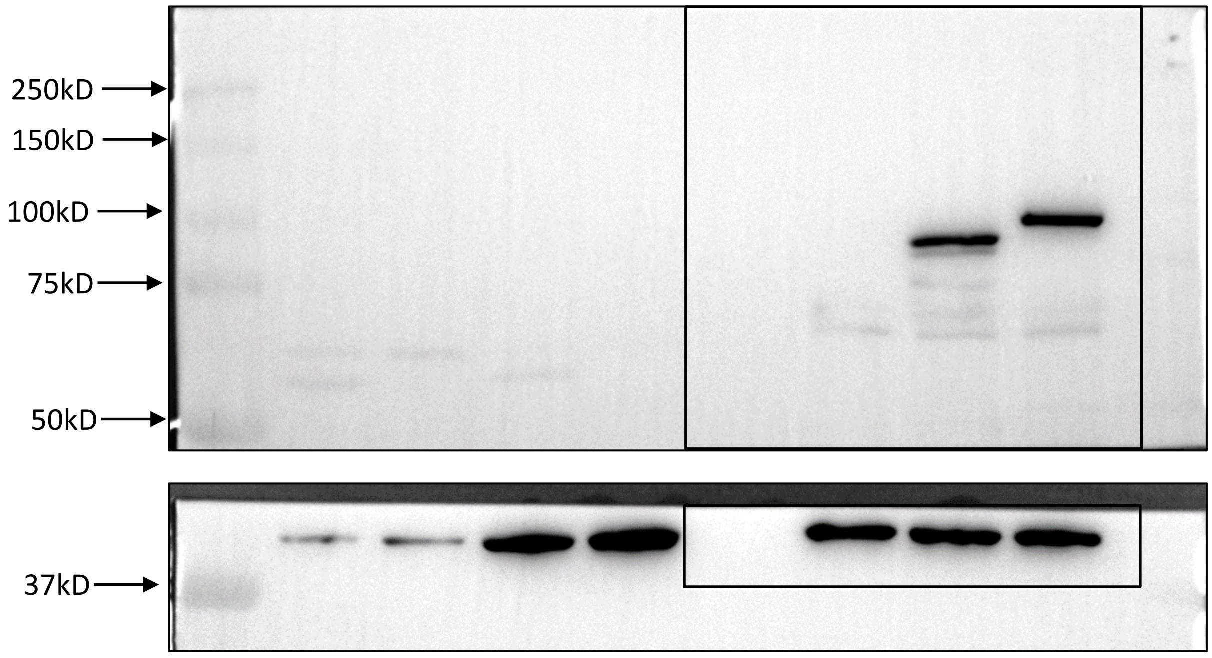

Supp Fig 1C

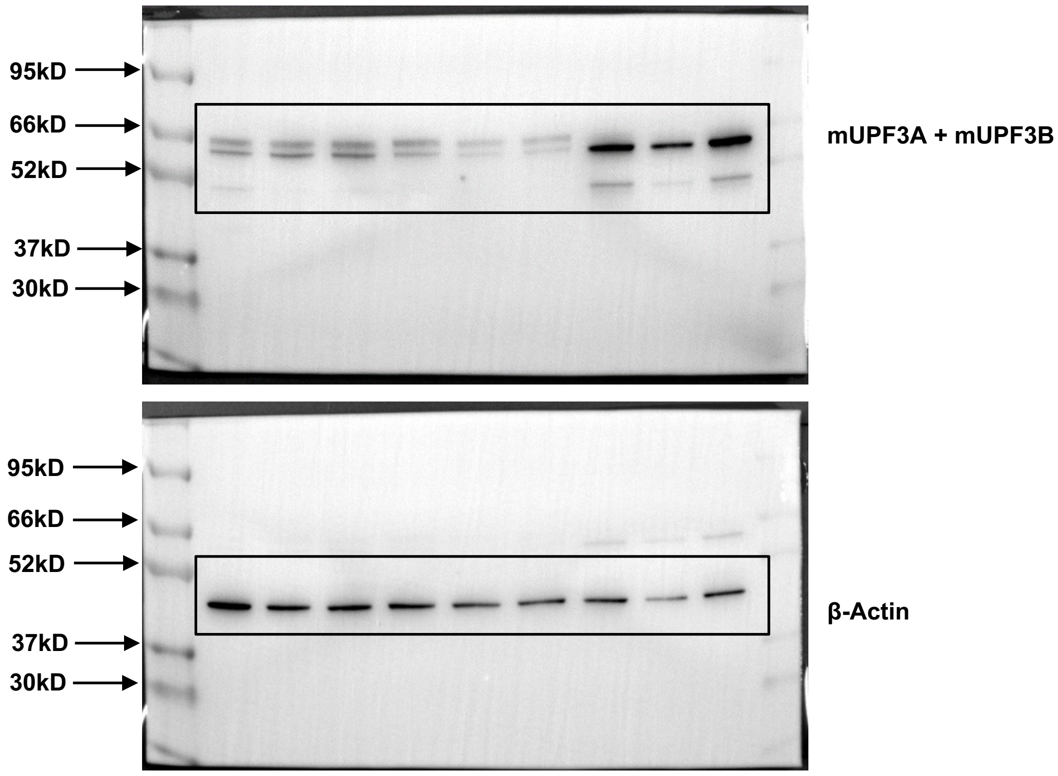

Related to Supp Fig 3

Supp Fig 3A

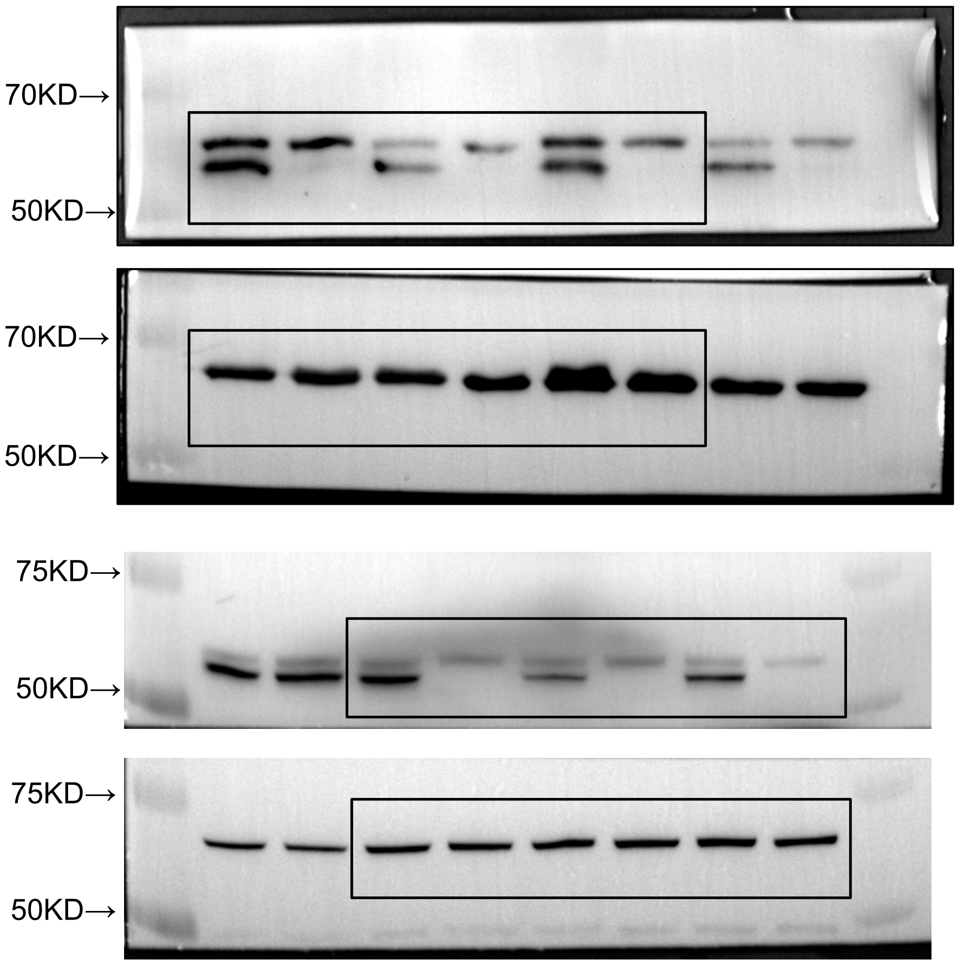

Related to Supp Fig 7

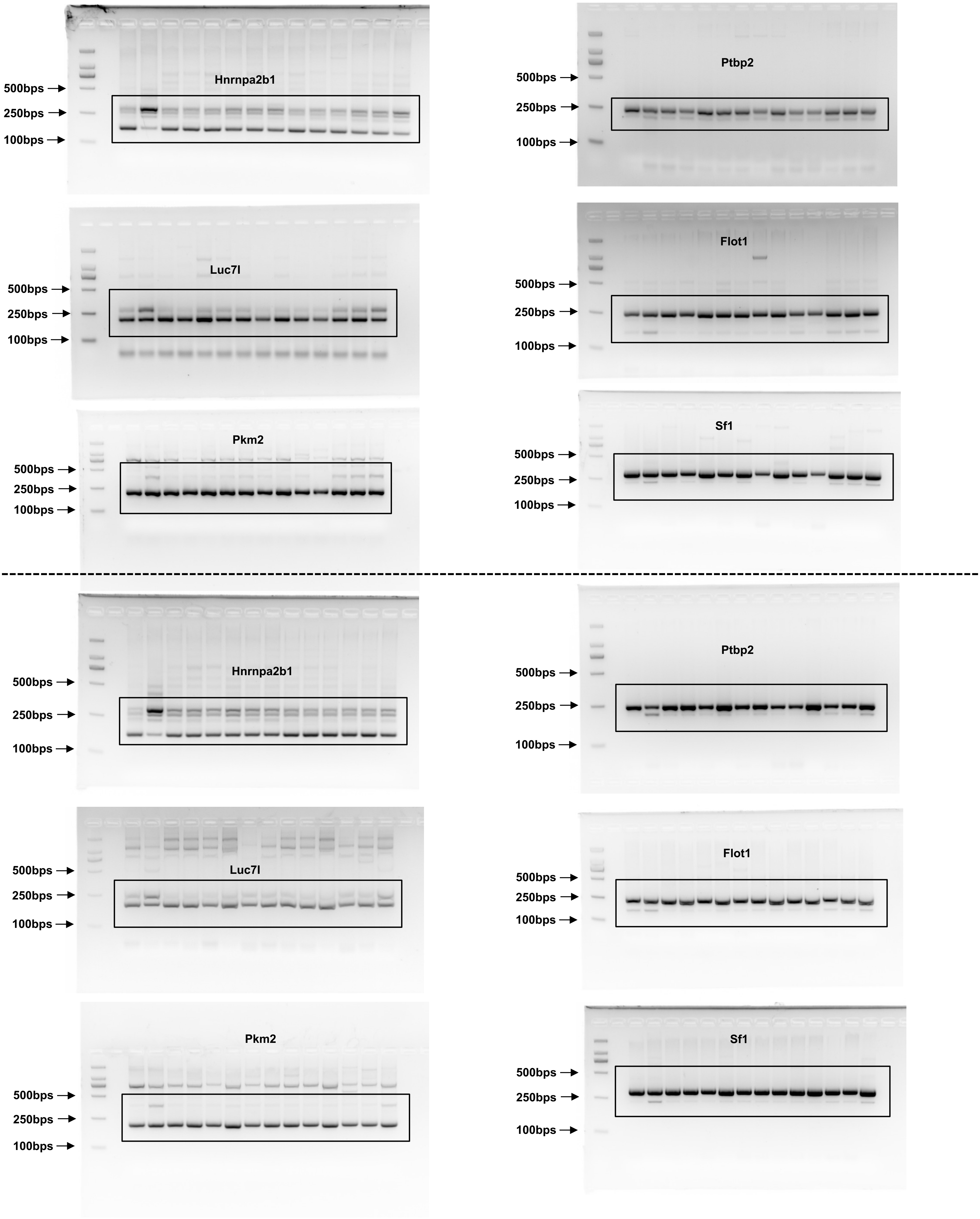

Related to Supp Fig 8

Supp Fig 8A

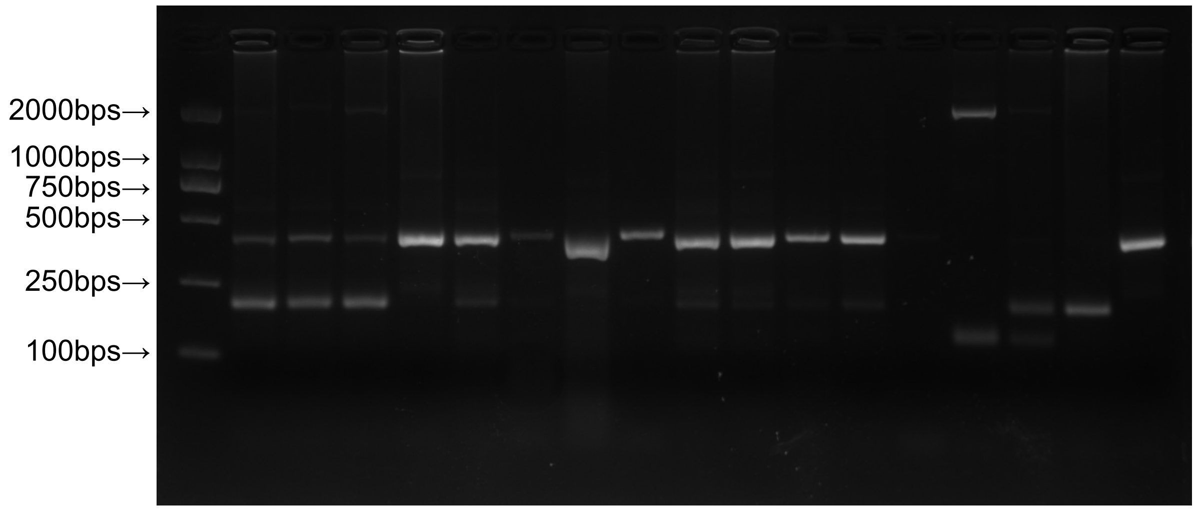

Supp Fig 8B

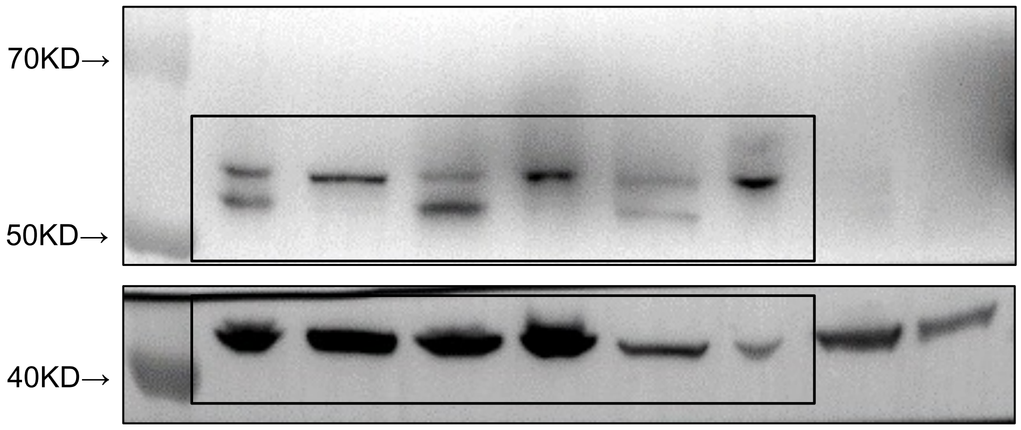

Related to Supp Fig 9

Supp Fig 9A

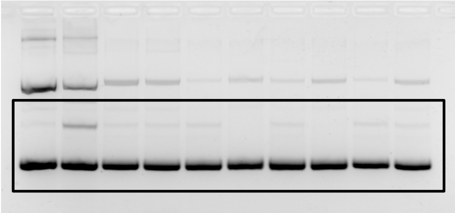

Pkm2

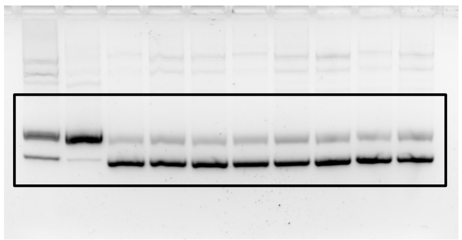

Eif4a2

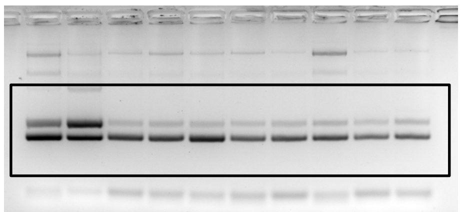

Luc7l

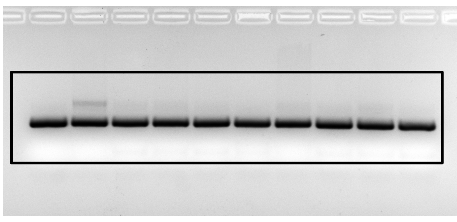

Snrpb

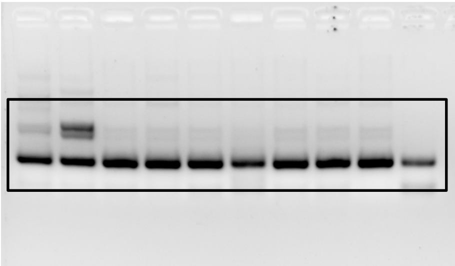

Rps9

Supp Fig 9B

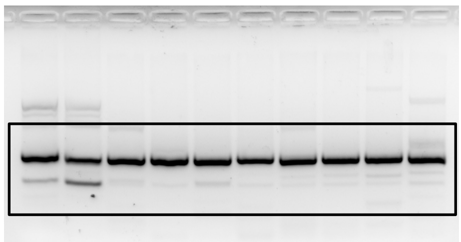

Alkbh3

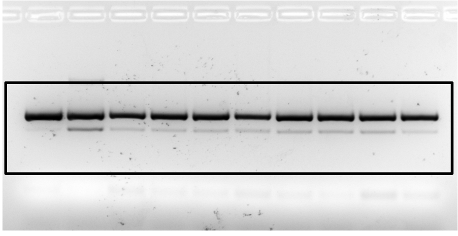

Sf1

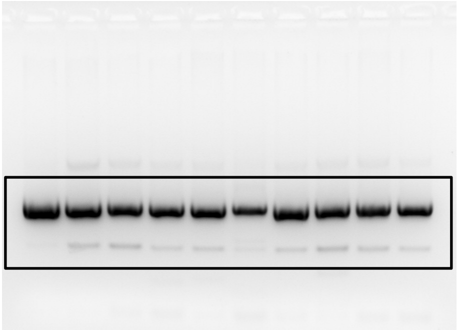

Nfyb

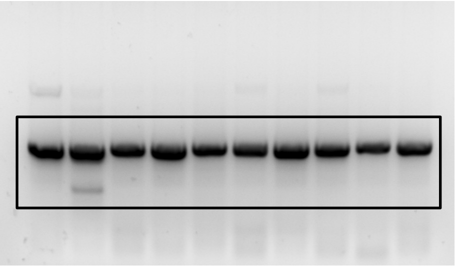

Slc38a2

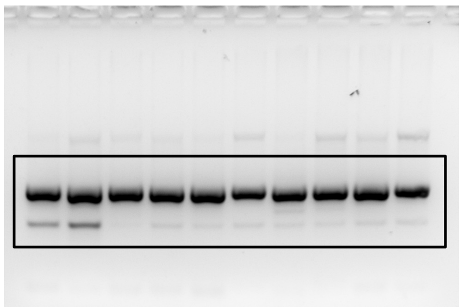

Flot1

Related to Supp Fig 9

Supp Fig 9C

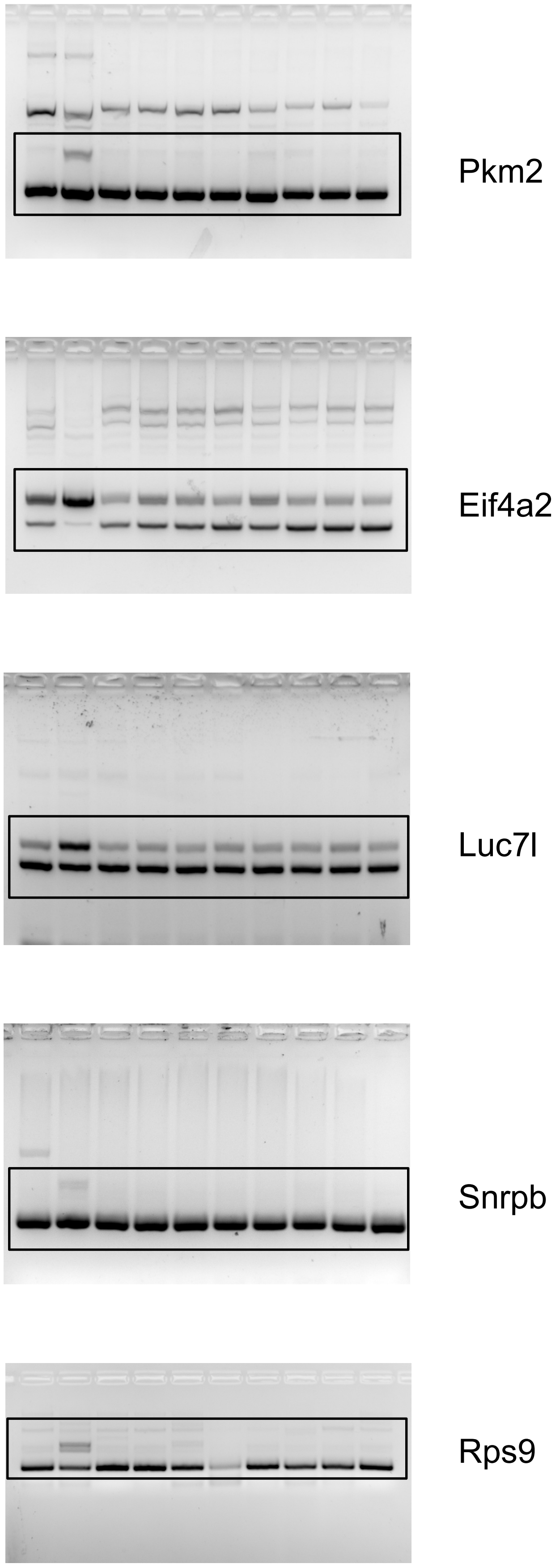

Supp Fig 9D

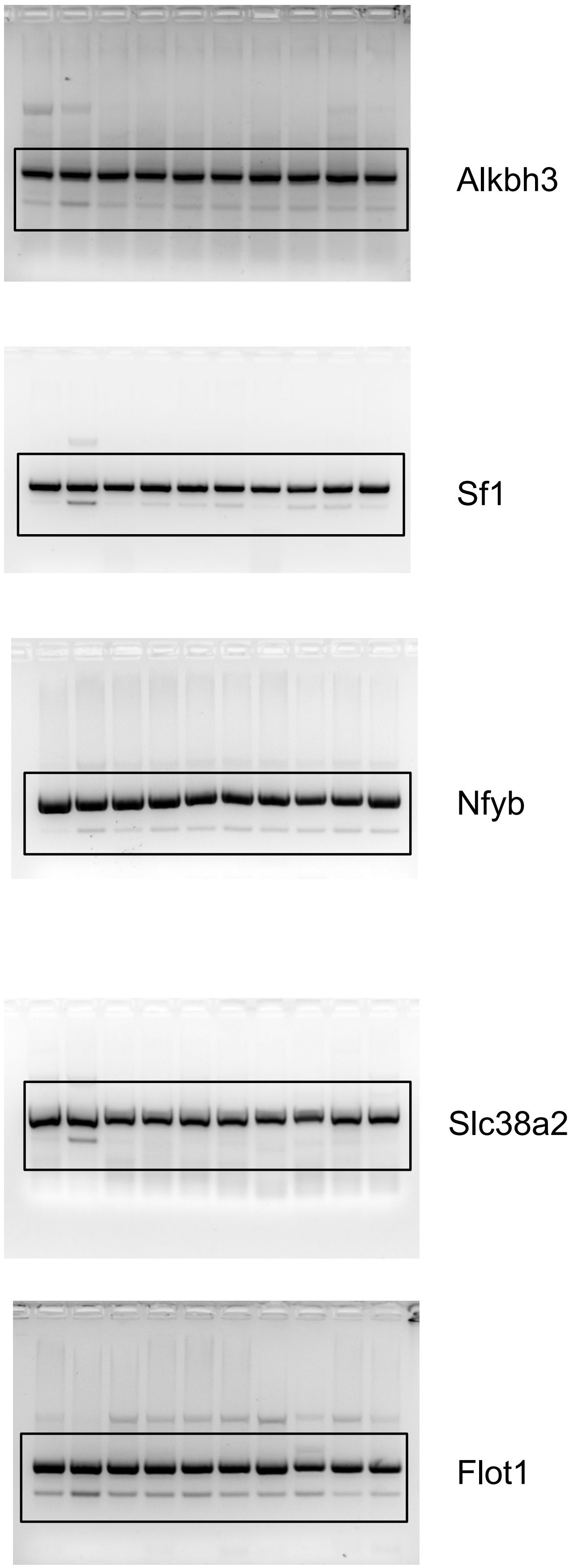

Supplement: Supplementary file 1 [file LSA-2022-01589_SdataF1_F3_FS1_FS3_FS7_FS8_FS9.pdf]
